# Role of face masks and ventilation rates in mitigating respiratory disease transmission in ICU

**DOI:** 10.1038/s41598-023-38031-x

**Published:** 2023-07-10

**Authors:** Venugopal Arumuru, Rajendra Kusuluri, Dnyanesh Mirikar

**Affiliations:** Applied Fluids Group, School of Mechanical Sciences, Indian Institute of Technology, Bhubaneswar, 752050 India

**Keywords:** Health care, Energy science and technology, Engineering

## Abstract

Indoor environments are major contributing locations where the respiratory virus transmission occurs. Higher air change rate (ACH) values (up to 12) have been recommended in hospital environments to reduce virus transmission. In the present study, the Large Eddy Simulation (LES) data of particle transport in a typical intensive care unit (ICU) is used to calculate the infection risk in close proximity interaction. Three different ACH (6, 9, 12) rates with face masks and one case with a healthy person wearing a face shield are considered. The average resident time of the droplets in the ICU is calculated to find the optimal ACH rate. Of the different types of masks analyzed in the present study, the triple-layer mask has shown the most resistance ($0 \%$ probability of infection) to the penetration of virus-laden droplets, while the single-layer mask has shown the highest risk of infection (up to $97 \%)$. The results show that the ACH rate has little effect on close proximity transmission. The ACH 9 case provided optimal value for the particle removal, while the ACH 12 has inferior performance to that of ACH 9. From an energy consumption view, our results recommend not using higher ACH in similar indoor environments. Inside indoor environments, it is advised to wear a three-layer face mask and face shield to reduce the risk of infection.

## Introduction

COVID-19 outbreak in the recent past has posed severe techno-economic challenges to humankind ranging from innovative strategic policies, technological developments, and changes in human behavior. However, the outbreak is now under control due to the rapid growth of vaccines and governmental policies related to combating the spread. In hospital environments, the exhaled virus-laden droplets from patients suffering from various diseases cause air contamination. Contaminated air carrying infectious pathogens can be transmitted to a healthy person by direct inhalation or deposition on mucous membranes. The risk of infection to susceptible people is dominated by the spatial and temporal distribution of droplet concentration inside the indoor environment. As the droplet size exhaled through coughing is small, the airflow patterns inside the room play a significant role in the dispersion of the droplets.

To calculate the risk of infection, using the Wells-Riley equation, Dai and Zhao^1^ reported the relationship between infection likelihood and ventilation rates and estimated the rate of ventilation required to keep the probability of infection below 1%. Agrawal and Bhardwaj^2^ calculated the likelihood of infection for a susceptible person close to an infected person and compared it to the super spreader. Liu et al.^3^ used large eddy simulations (LES) simulations to get deeper into the fluid dynamics of the puff ejected by sneezing and coughing. The theoretical model predicts the properties of the respiratory puff, including droplet size, velocity, and the distance they travel. Zhao et al.^4^ used LES data to predict the effect of the face shield on the source of coughing. The droplet penetration length is reduced by $\sim 74\%$ when the emitter wears a face shield. Vadlamudi et al.^5^ analyzed the penetration and aerosolization of the cough droplets through the face mask to consider the effect of porous size, thickness, and porosity. They observed that all droplet sizes penetrate the face mask beyond a certain velocity. Sharma et al.^6^ conducted an experimental study on the secondary breakup of the droplets impacting the face mask. Different types of masks with various layers have been considered in the study. A large-sized ($>250 \mu m$) droplet break-ups into small diameter droplets ($<100 \mu m$), which can remain airborne for a significant time as compared to emitted large droplets. Three-layer masks proved to be influential among the tested, which can block the ejected droplets to a great extent.

An Intensive Care Unit is a typical indoor environment where a healthcare professional routinely interacts with an infected person. The working environment must be optimized for the safety and well-being of healthcare staff. An effective ventilation strategy is required to remove pathogens from such indoor environments. A modern mechanical ventilation system’s tasks include maintaining human comfort (temperature and relative humidity within acceptable limits), air filtration, managing air distribution, and venting the air out of the space via air exchange^7–9^. The clinical significance of heating, ventilation, and air conditioning (HVAC) is briefly explained by Saran et al.^7^. Saran et al.^7^ suggested that ACH for a standard ICU room in most countries is six. In the United States of America (USA) and India, guidelines for developing and planning ICUs similarly prescribe an ACH value of six^10^. Bhattacharyya et al.^11^ used CFD simulations to establish optimum airflow patterns for distributing sanitizer in the isolation chamber. The study aimed to determine the best mixing features between a sanitizer flow and a ventilation system flow. The droplet dispersion in a two-bed hospital ward has been studied by Ren et al.^12^ with different ventilation layouts. Results indicated the outlet be installed at the location where large droplets ($> 45\mu m$) deposit. According to Akagi et al.^13^, $4.4\%$ of the expelled particles from sneezing enter inside a face shield, indicating the likelihood of virus exposure. Komperda et al.^14^ examined the danger of contamination in a big dentistry clinic statistically, indicating the importance of vent position relevant to people. Reference^15^ results show that there is a certain threshold beyond which the effect ventilation rate and the physical distance doesn’t have significant effect on the transmission. They have found minimal physical distance and maximum ventilation rate to be maintained to minimize the infection risk for different types of movements. Qian et al.^16^ investigated particle dispersion in a two-bed hospital ward using various ventilation methods due to their effective particle removal capabilities. They advocated mixing and downward ventilation systems for multi-bed hospital wards. Khankari^17^ used CFD simulations to investigate the ventilation patterns in a typical single-bed ICU room. The numerical study demonstrates the significance of the placement of the vents and ventilation rate on the particle route for various air diffuser and exhaust configurations in an isolation room. Borro et al.^18^ used CFD simulations to investigate the function of air conditioning in virus-laden particle diffusion in the hospital’s waiting area and recovery room. According to their findings, boosting the ventilation inlet rate lowers particle concentration and increases turbulent air transport, resulting in virus-laden particles spreading quickly and over great distances. It’s worth noting that the majority of the studies focused on various ventilation configurations, and it’s advised that air change rates be increased^19–21^.

Interaction with patients suffering from respiratory disease is pervasive in health care hospitals, ICUs, and isolation wards. Such interactions can effectively transmit the virus to a healthy person if proper safety norms are not followed. Hence, analyzing such scenarios is of great interest and determining the factors that can be controlled to reduce the risk of infection. The face mask is used as a source control measure that effectively reduces the infection rate. Recently, significant efforts have been documented to establish face mask effectiveness under different scenarios. Most of these studies focused on the efficacy of face masks in preventing the escape or trapping of droplets from an infected host. However, studies are scarcely reported to understand the effectiveness of a face mask in avoiding infection to a healthy person wearing a face mask. Face mask effectively blocks the escape of large diameter particles, and smaller diameter particles that can remain suspended may leak from the mask, the extent of which depends on the type of the mask. It is worth noting that a health care person must frequently interact with an infected person in an ICU or isolation ward. This interaction typically occurs in proximity as the healthcare person may have to inspect or assist the infected person. As per guidelines, the healthcare person needs to wear a mask. In the present study, we consider a typical scenario where the infected person without a mask cough when the healthcare person is in close proximity. Under this scenario, we evaluate the risk of infection to the healthcare person wearing a mask. We considered the influence of the type of mask (singly-layer, two-layer, and three-layer) and the impact of ACH on the risk of infection, such a realistic and relevant study is seldom reported in the literature and has a particle relevance in the present scenario. Various air change rates (6, 9, 12) are used in the current scenario and compared to analyze the effect of the droplets deposition onto a susceptible person’s face mask. The case where a healthy person wearing a face shield has also been numerically simulated. The probability of infection is evaluated for various types of masks and face shields used by a susceptible person. Such a study is important to determine whether a higher ACH is recommended to reduce the risk of infection because the higher ACH conditions will result in larger energy consumption for the ventilation system.

## Methodology

### Model description

#### Geometry

**Figure 1:**
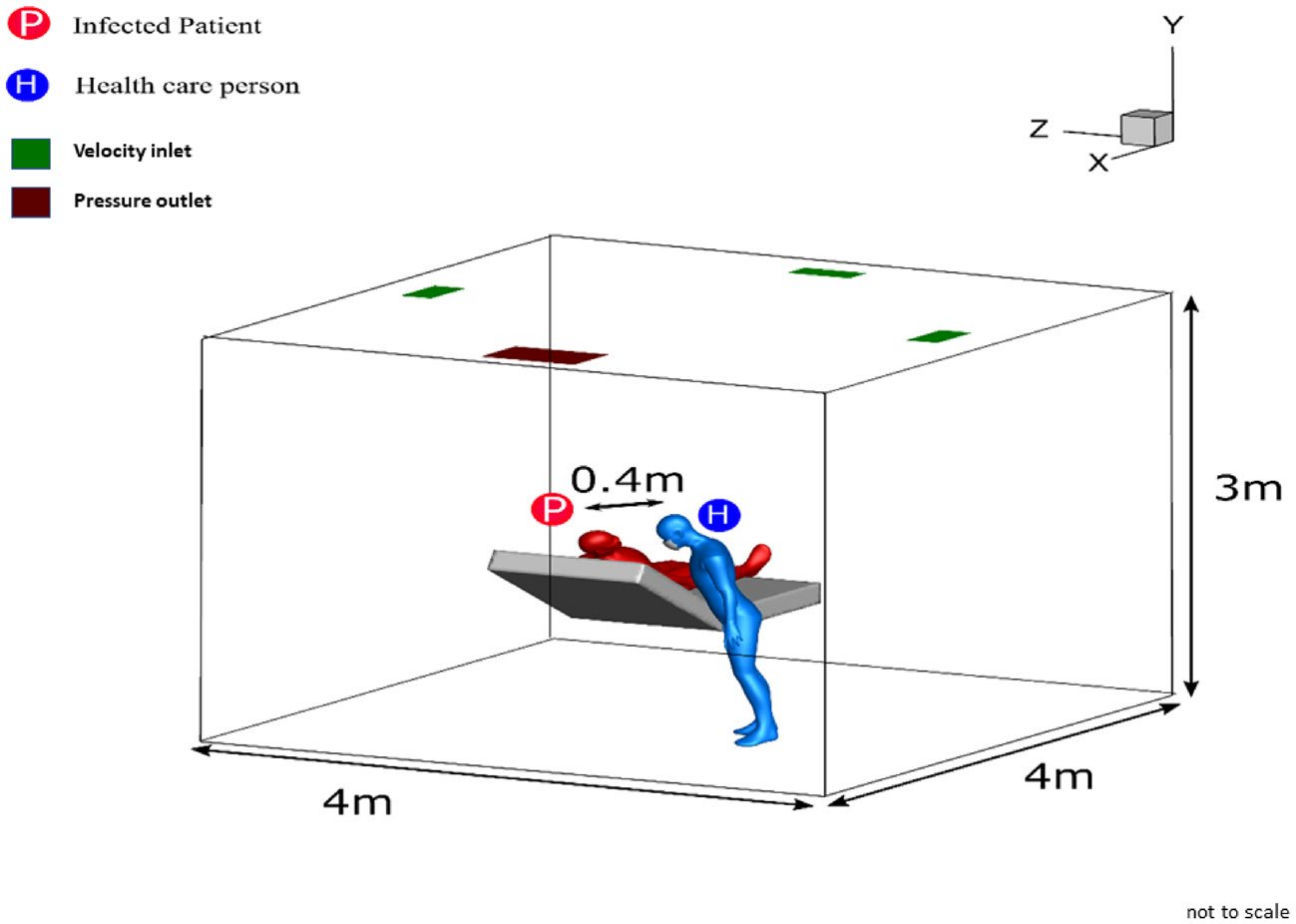
Geometry: ICU room with the interaction between health care person and patient.

A typical three-dimensional indoor space (ICU room) with dimensions of 4 m × 4 m × 3 m (length × width × height) is selected for the analysis as suggested by Rungta^22^ which is shown in Fig. 1. The computational domain consists of a healthy healthcare person and an infected patient lying on the bed. An air conditioning system is used on the top wall of the ICU room using three inlet vents, each with an area of $0.4\times 0.2 {m}^{2}$ and one outlet vent of $0.4\times 0.6 {m}^{2}$ area, as shown in Fig. 1. The area of the patient’s mouth is $3.124 {cm}^{2}$ which is considered a cough source. In the interaction scenario, the healthcare personnel is slightly inclined in a way that resembles regular interaction between patient and healthcare worker as shown in Fig. 1. The facial orientation is in such a way that they interact. The air enters the room at a volumetric flow rate of $288\, {m}^{3}/h(ACH-6), \,432 \,{m}^{3}/h(ACH-9), \,and\, 576 \,{m}^{3}/h \,(ACH 12)$ from the inlets. The ventilation outlet positioning is selected above the patient’s head as suggested by Khankari^17^. His study with various ventilation layouts indicated that the outlet vent location just behind patients head allows particles to exit the room without significant recirculation and entrainment back into the supply airstream.

#### Numerical modeling and boundary conditions

The computational domain in the current problem is modeled as two coupled phases. The first is a continuous phase that contains the fluid, and the second is the discrete phase, which accounts for the droplets released in the event of coughing. The Eulerian–Lagrangian approach solves the domain equations in the Large Eddy Simulation (LES) framework. The Eulerian approach is incorporated to solve the continuous phase equations, and the discrete phase equations are solved by the Lagrangian method to obtain the unsteady movement of the discrete phase particles. The equations are solved by ANSYS FLUENT commercial software package. Without considering the Discrete phase sources, a steady-state flow is established inside the ICU room to maintain a mean airflow rate with a relative humidity of 50% before injecting the droplets, and unsteady simulations with coughing are performed after that using appropriate time steps. The boundary conditions for the continuous and discrete phases are specified in Tables 1 and 2.

**Table 1:** Continuous phase boundary conditions.

Parameters	Values
Cough airflow (Mouth of the patient)	Time-varying velocity profile^23^
Relative humidity	97%^24^
Velocity at inlet (Inlet Vents)	0.333 m/s [ACH 6]
Mouth surface area	3.124 ${cm}^{2}$^25^
Outlet pressure (Outlet vents)	101,325 Pa
Cough air temp	312.5 K^26^
Room temperature and relative humidity (RH)	293 K and 50%
Temperature of the patient & health care person, respectively	312.5 K, 309.5 K^26^

**Table 2:** Boundary conditions for discrete phase.

Parameters	Values
Droplet velocity	8.5 m/s Single cough^25^
Half cone angle (Injection)	15^0^^23^
Temperature of the droplets expelled in coughing	312.5 K^25^
Droplet diameter distribution	$1-300 \mu m$^27^ fitted with Rosin–Rammler diameter (RRD) distribution
Injection time	0.4 for single cough^23^
Mass of droplets injected	2.87 × 10^−7^ $kg$
Discrete phase boundary conditions	Trap: The particle trajectory calculations are stopped. This condition is applied to all solid surfaces like walls, persons, and bed Escape: The droplet is assumed to escape the simulation. This condition is applied at all the vents

The continuous phase in the computational domain has phenomena like fluid flow, heat transfer, turbulent dispersion, and species transport. Two-way coupling between the continuous and discrete phases is employed in the present study. The continuous phase equations are solved first, and then the source terms from the discrete phase equations solution are added to the continuous phase to ensure the coupling between the two phases.

Large Eddy Simulation (LES) is employed to model the continuous phase using Smagorinsky-Lily sub-grid scale model. The flow is assumed incompressible, unsteady, and turbulent. The commercial software ANSYS FLUENT 19.0 was used to solve the corresponding governing equations^28^. The filtered continuity and momentum equations solved are given by

1 $$\begin{array}{c}\frac{\partial {\overline{u} }_{i}}{\partial {x}_{i}}=0,\end{array}$$

2 $$\begin{array}{c}\frac{\partial \left(\rho {\overline{u} }_{i}\right)}{\partial t}+\frac{\partial \left(\rho {\overline{u} }_{i}{\overline{u} }_{j}\right)}{\partial {x}_{j}}=\frac{\partial {\sigma }_{ij}}{\partial {x}_{j}}-\frac{\partial \overline{p}}{\partial {x }_{i}}-\frac{\partial {\tau }_{ij}}{\partial {x}_{j}}.\end{array}$$

The over-bar corresponds to locally-averaged filtered variables. The laminar stress tensor is

3 $$\begin{array}{c}{\sigma }_{ij}=\mu \left(\frac{\partial {\overline{u} }_{i}}{\partial {x}_{j}}+\frac{\partial {\overline{u} }_{j}}{\partial {x}_{i}}\right).\end{array}$$

The subgrid stress accounting for the unresolved scales contribution is defined by:

4 $$\begin{array}{c}{\tau }_{ij}=\rho \left({\overline{u} }_{i}{\overline{u} }_{j}-{\overline{u} }_{i}{\overline{u} }_{j}\right).\end{array}$$

It is modeled using the Boussinesq hypothesis

5 $$\begin{array}{c}{\tau }_{ij}-\frac{1}{3}{\tau }_{kk}{\delta }_{ij}=2{\mu }_{t}{\overline{S} }_{ij},\end{array}$$

where the resolved strain rate is defined by

6 $$\begin{array}{c}{\overline{S} }_{ij}=\frac{1}{2}\left(\frac{\partial {\overline{u} }_{i}}{\partial {x}_{j}}+\frac{\partial {\overline{u} }_{j}}{\partial {x}_{i}}\right).\end{array}$$

The subgrid scale turbulent eddy-viscosity is obtained from

7 $$\begin{array}{c}{\mu }_{t}=\rho {L}_{s}^{2}\sqrt{2{\overline{S} }_{ij}{\overline{S} }_{ij}}.\end{array}$$

${L}_{S}$ is calculated using

8 $$\begin{array}{c}{L}_{s}=\mathrm{min}\left(\kappa d,{C}_{s}{V}^{1/3}\right),\end{array}$$

where $\kappa$ is the Von Karman constant, $d$ is the distance closest to the wall, ${C}_{S}=0.12$ is the Smagorinsky constant dynamically calculated based on the information included in the resolved scales of motion^29,30^ and $V$ is the volume of the computational cell. The filtered energy equation is written as:

9 $$\begin{array}{c}\frac{\partial \overline{T}}{\partial t }+{\overline{u} }_{j}\frac{\partial \overline{T}}{\partial {x }_{j}}=\frac{\partial }{\partial {x}_{j}}\left(\frac{\nu }{\sigma }\frac{\partial \overline{T}}{\partial {x }_{j}}\right)+\frac{\partial {q}_{j}}{\partial {x}_{j}},\end{array}$$

where $\sigma$ is the Prandtl number and the subgrid-scale heat flux is defined as

10 $$\begin{array}{c}{q}_{j}={\overline{u} }_{j}\overline{T }-\overline{{U }_{j}T},\end{array}$$

and is calculated using the subgrid-scale turbulent Prandtl number

11 $$\begin{array}{c}{q}_{j}=\frac{{v}_{t}}{{\sigma }_{t}}\frac{\partial \overline{T}}{\partial {x }_{j}}.\end{array}$$

The turbulent Prandtl number is estimated by applying the dynamic procedure to the subgrid-scale flux^29,30^. Droplets are transported by the movement of air and water vapor mixture. External forces influence the droplet trajectories. Droplet particles are tracked by solving Newton’s second law of motion: inertial force equals the sum of all external forces acting on the particle. In the present scenario, gravitational, drag, and lift forces are considered, and coupling between continuous and discrete phases is performed. The coagulation effect is neglected for this diameter range; hence these effects are not considered for the following study. The turbulence dispersion of the droplets is tracked by the Discrete random walk model (DRW), which is done by stochastic tracking, considering instantaneous fluctuations in the droplet trajectories. Many authors have successfully used the DRW model^20,31–33^. The residuals of continuity, conservation of momentum, energy, and species transport were kept below ${10}^{-5}$ as a necessary condition. The mass flow rate at the ventilation outlet is monitored as a sufficient condition to arrive at a steady-state solution. Other numerical modeling, continuous and discrete phase boundary conditions associated with various surfaces, and equations used to model heat and mass transfer are the same as modeled in our previous study^21^.

### Model validation and mesh independent study

**Figure 2:**
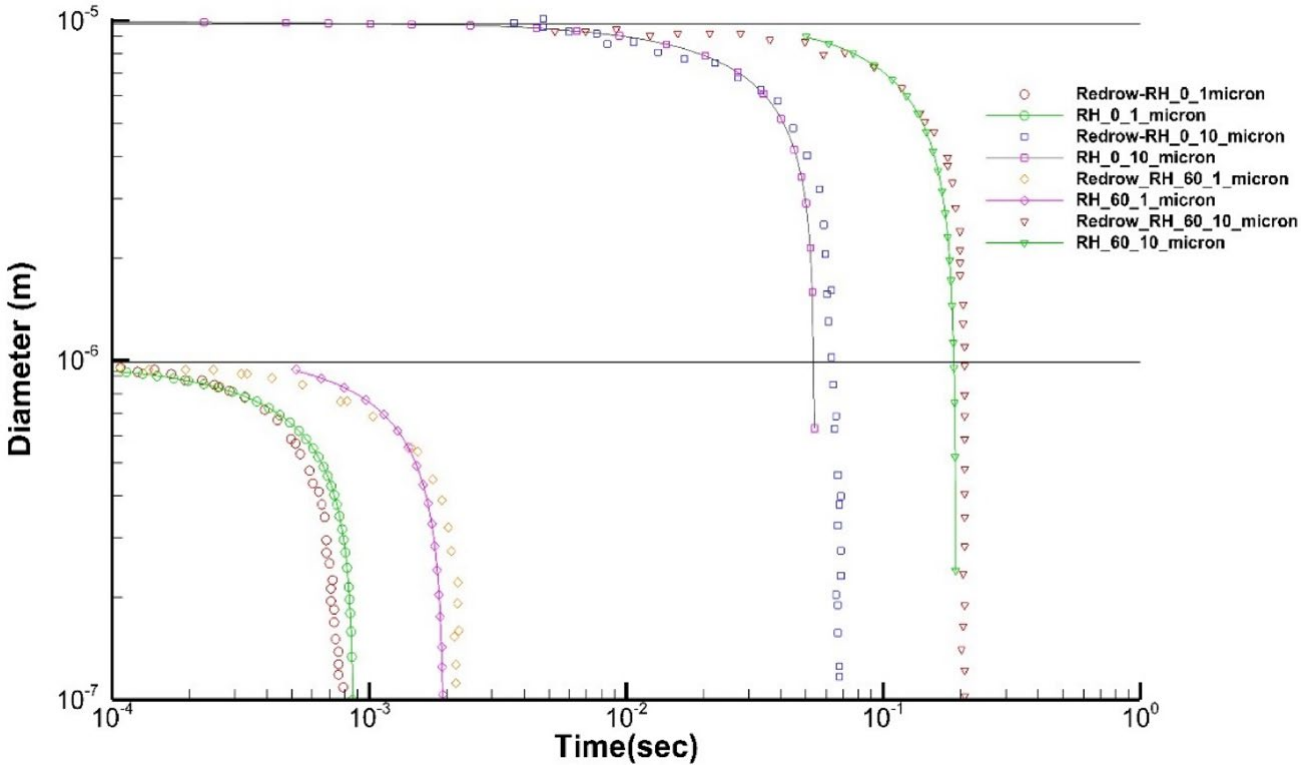
Evaporation time of droplets at a different relative humidity.

The Eulerian–Lagrangian discrete phase model (DPM) is validated with existing literature data for the evaporation time of the water droplets for two different diameters. As shown in Fig. 2, we recreated Redrow et al.’s^34^ numerical setup and plotted the evaporation time for a pure water droplet with diameters of 1 and 10 µm to compare their results for two distinct humidity levels of $RH=0 \%$ and $RH=60 \%$.

**Figure 3:**
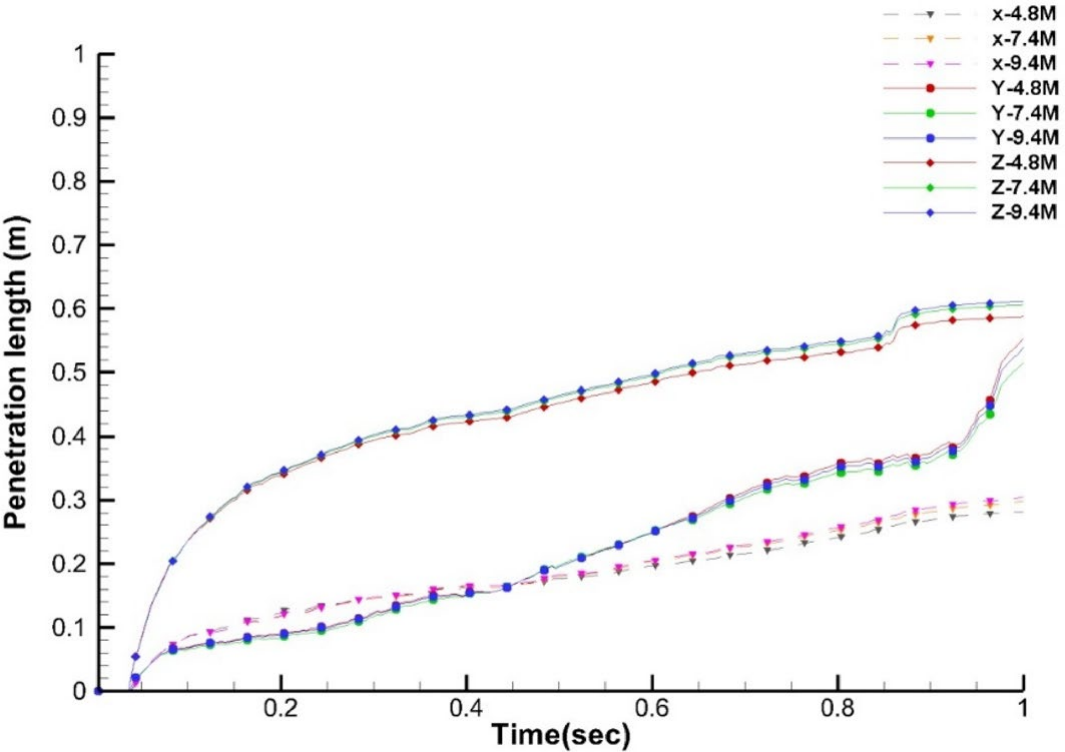
Mesh independence penetration length for the interacting geometry.

**Figure 4:**
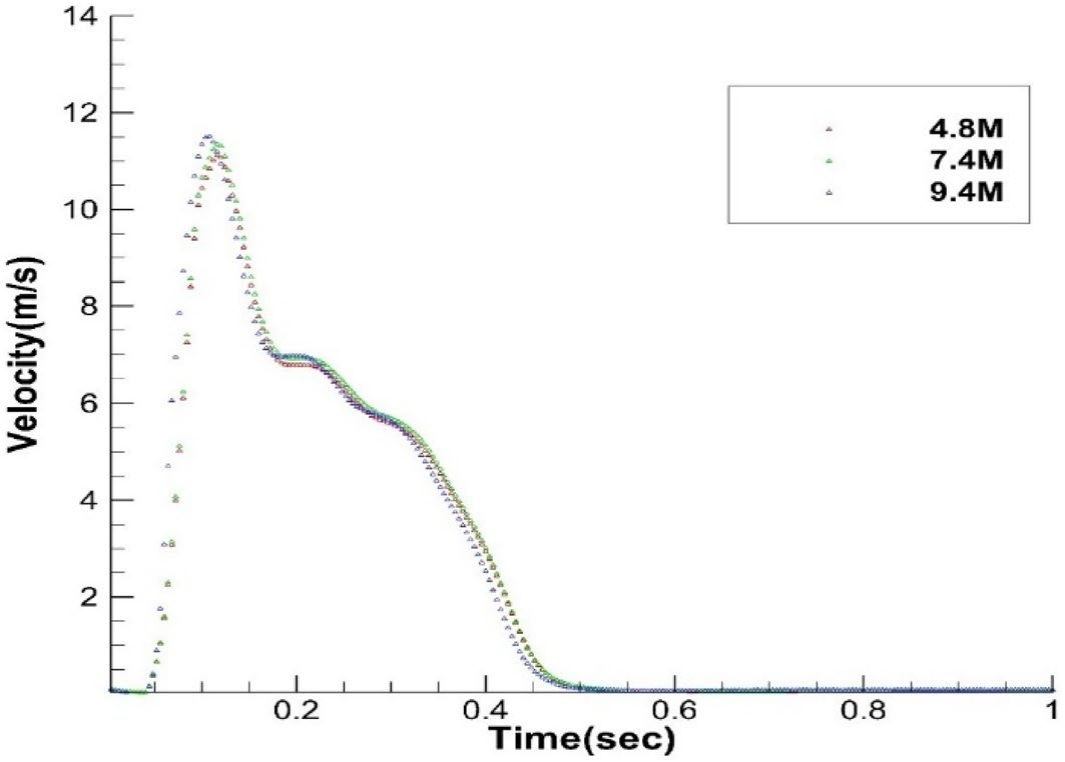
Mesh independence for continuous phase.

**Figure 5:**
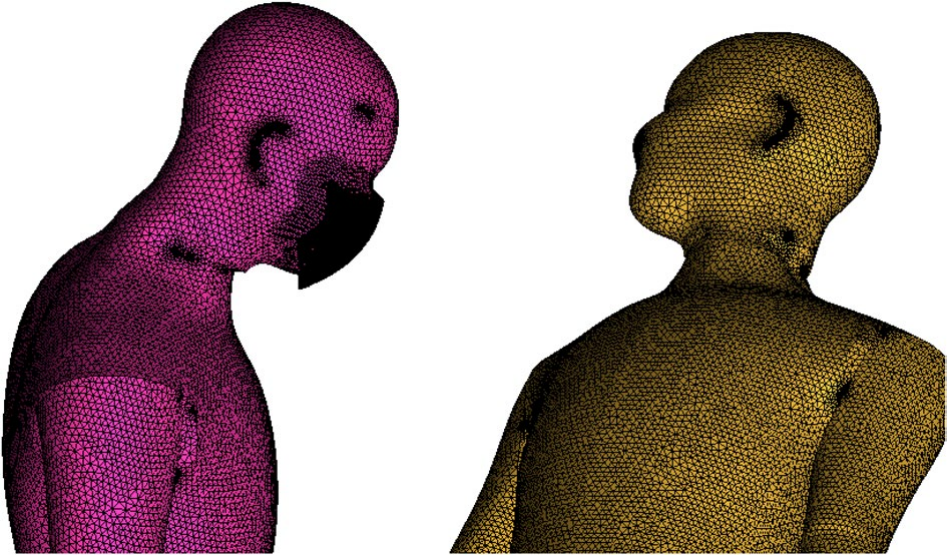
Close-up view of the mesh considered in the present study.

The mesh independence is carried out using three different mesh sizes, as reported in Table 3. The velocity of the continuous phase at a point in the path of ejected cough is compared for three different mesh sizes. The penetration length^25^ in x, y, and z directions for the discrete phase is compared for all three mesh sizes to check mesh independence. Figure 3 shows the penetration distance of the discrete phase. Figure 4 shows the continuous phase’s velocity variation for all three meshes at two points. It is observed from Figs. 3 and 4 that the variation of results for all three mesh sizes is not significant, and hence medium, size mesh (M2) with 7.4 × 10^6^ elements is employed for the simulations for standard ICU scenarios. A close-up view of the mesh generated on the human body is shown here for reference in Fig. 5.

**Table 3:** Number of elements in different mesh sizes for interaction geometry.

Mesh size	Number of elements
Coarse mesh [M1]	$4.8\times {10}^{6}$
Medium mesh [M2]	$7.4\times {10}^{6}$
Fine mesh [M3]	$9.4\times {10}^{6}$

## Results and discussion

### Vortex evolution of cough

**Figure 6:**
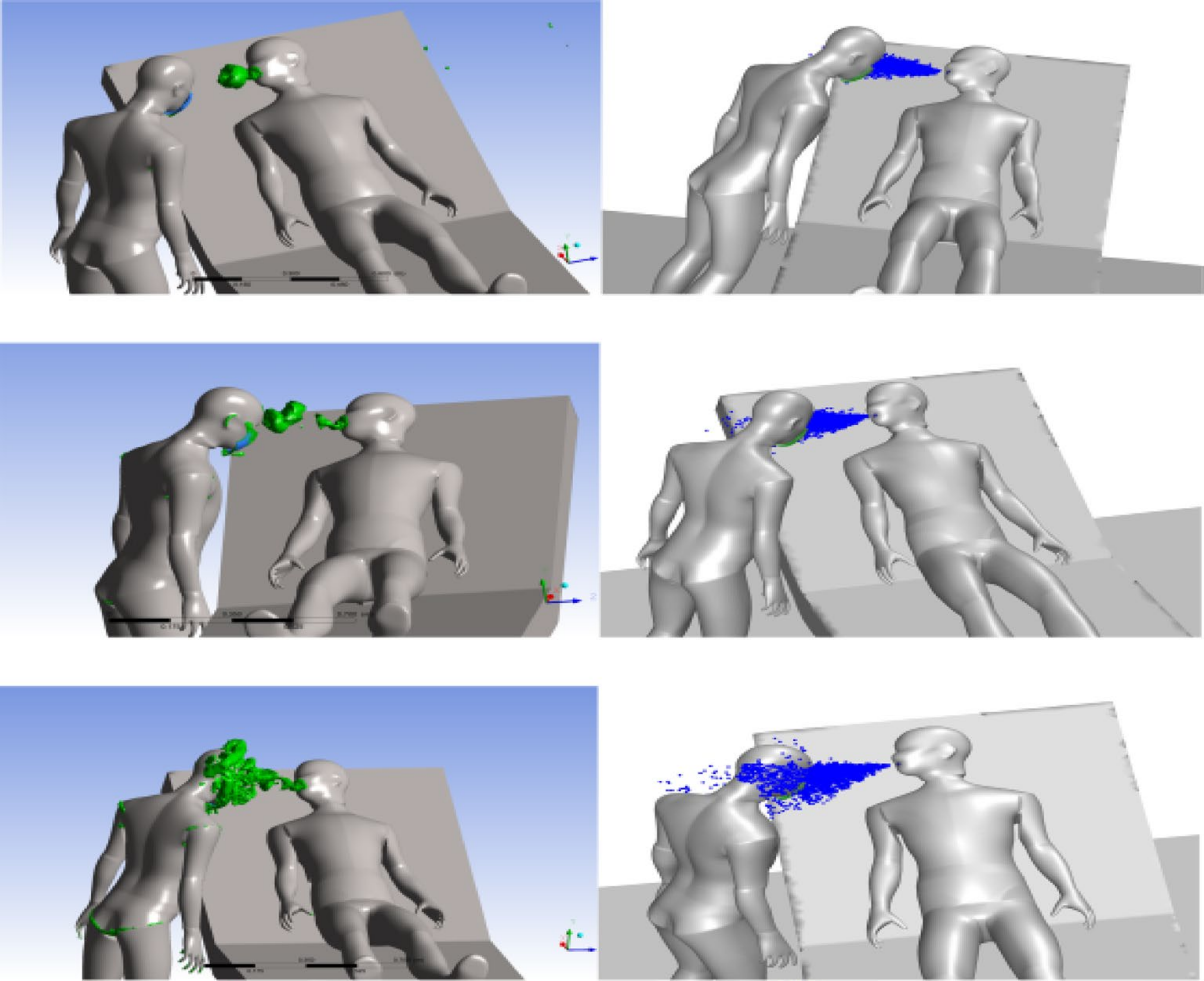
(Left): Evolution of the vortex generated (q-criterion = 0.001) due to coughing at t = 0.1, 0.15, 0.3 s from top to bottom. (Right): particle tracks at the same times at ACH-9.

A cough event from the patient generates a vortex structure in front of the patient, as shown in Fig. 6. The vortex structure formed during cough breaks and evolves in front of the patient, slowly diffuses, and impinges onto the health care person’s face taking the droplets released from the cough and depositing them on the face.

**Figure 7:**
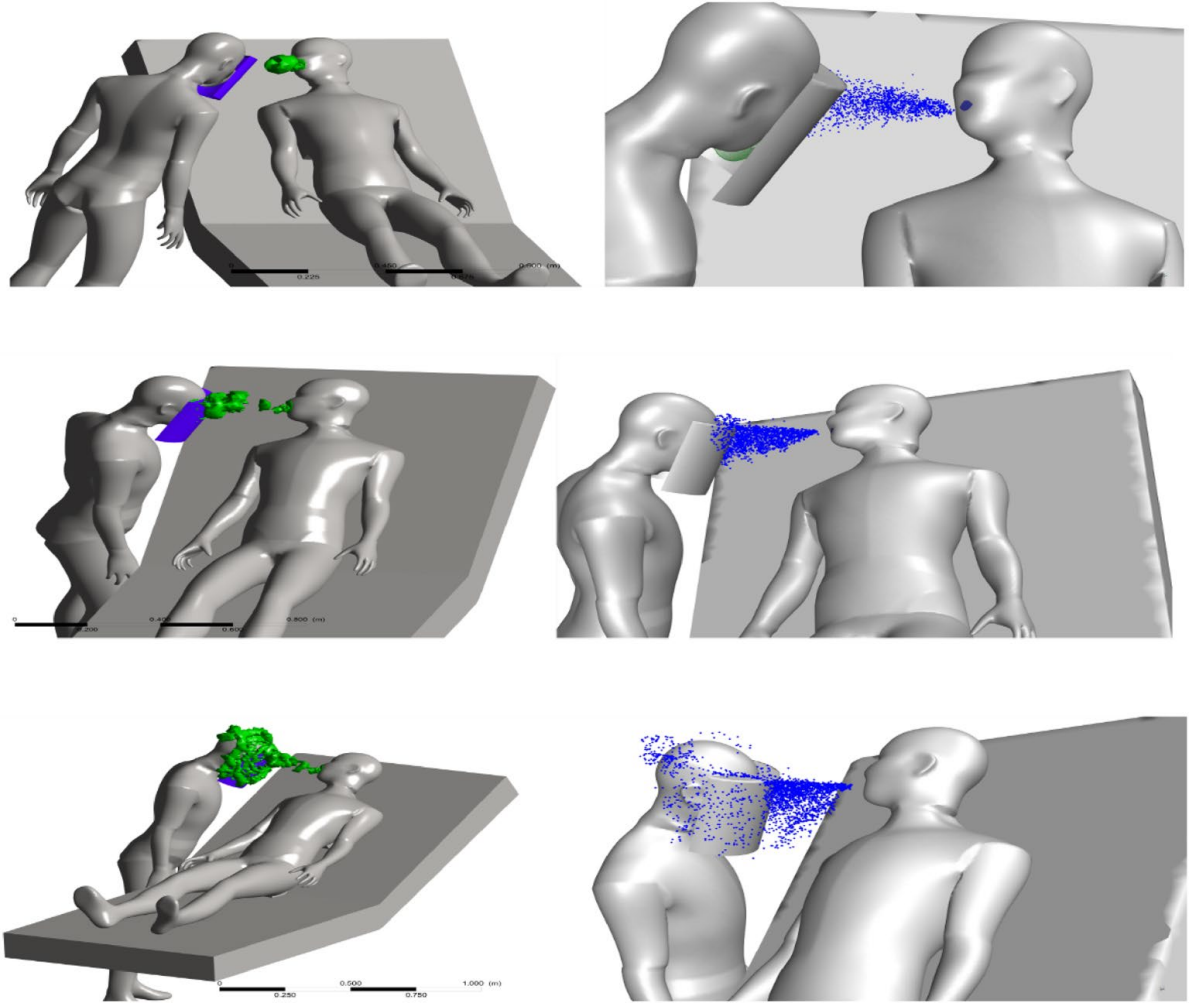
(Left): Evolution of the vortex generated (q-criterion = 0.001) due to coughing at t = 0.1, 0.15, 0.3 s from top to bottom. (Right): particle tracks at the same times at ACH-6 with a face shield.

Figure 6 shows the vortex structure evolution for ACH-9 at various times during cough and gives an insight into how this vortex structure breaks in addition to the particle evolution during this period. The variation of the cough evolution and vortex structure evolution for various air change rates is not significantly altered due to an increase in air change rate. It happens because the magnitude of the velocity field between the health care person and patient is not significantly changed for all the cases with different ACH. When a face shield is used in addition to the mask, a large number of droplets impinge onto the face shield, as shown in Fig. 7. None of the droplets released from the cough event enter the region near the mouth of the health care person, which is the best scenario out of all the cases explored in the current study. The current position of the healthcare person, ventilation location and rates does not allow any particles to reach inside the face shield, for the different simulations performed in the study.

**Figure 8:**
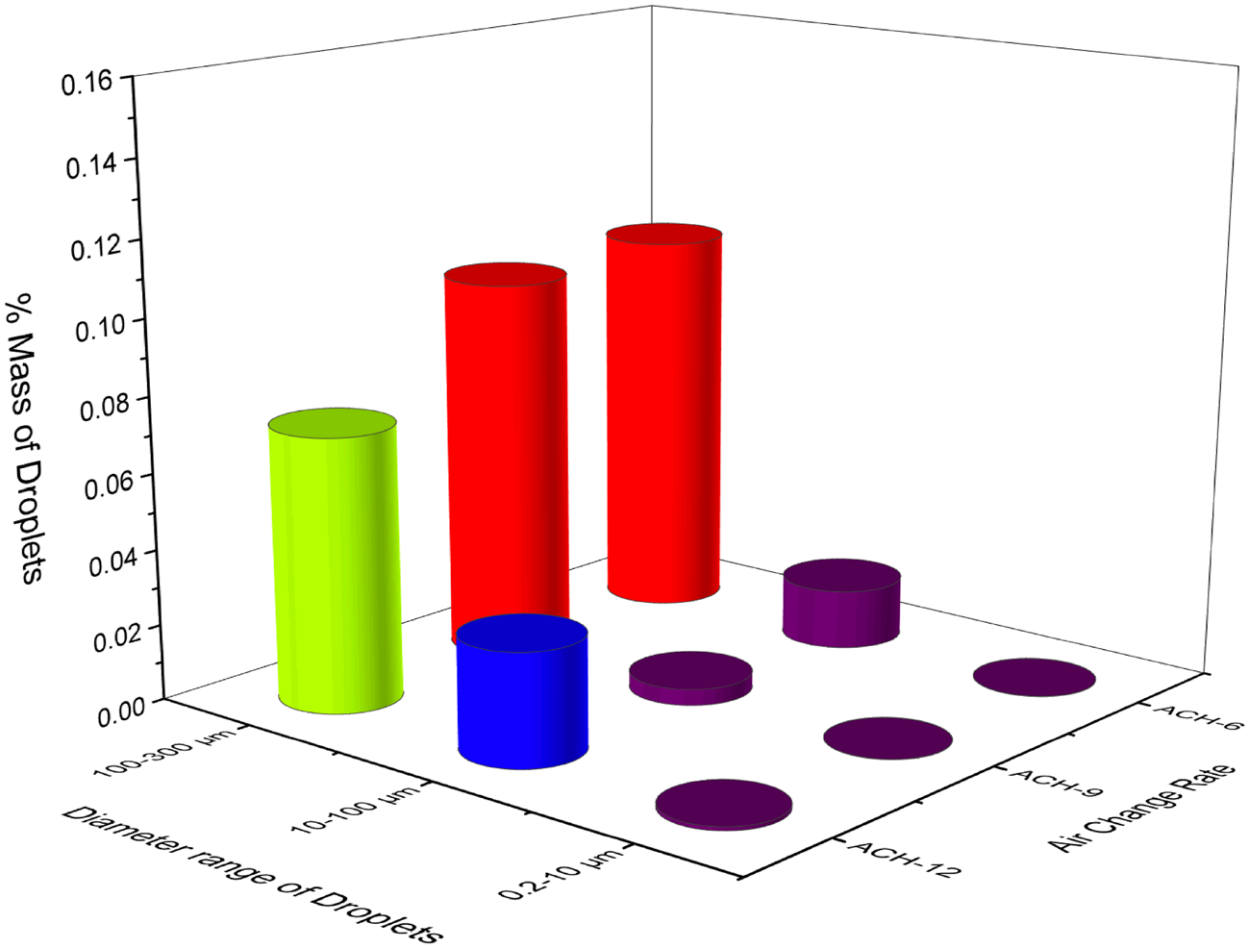
Mass of droplets inhaled (% of injected) for various ACH and different mask types.

Droplets released from the cough are consolidated into three different droplet ranges from $0.2-10 \mu m$, $10-100 \mu m,$ and $100-300 \mu m$. For different ACH rates, the mass of droplets deposited onto the face mask is shown in Fig. 8. Among all three cases, $\sim 10 \%$ of injected mass from coughing is deposited onto the face mask. The percentage of injected mass deposited on the face mask is maximum for the diameter range of $100-200 \mu m.$ While the the smaller droplets are advected along the airflow, the larger droplets are deposited on nearby surfaces. Medium-sized droplets follow the puff ejected by the coughing source and are found to be deposited directly onto the substance coming in the path.

### Estimation of the infection risk

The risk of infection is the probability that a person sharing a space with the infected person indoors will get infected with the disease. The risk of infection via airborne particles is given by a Poisson’s distribution, according to the original model proposed by Wells and Riley^35^, where the infection probability $P$ is dependent on the total “infectious quanta” of the pathogen inhaled ${n}_{quantum}$,

12 $$\begin{array}{c}P=1-{e}^{{n}_{quantum}}.\end{array}$$

To accurately calculate the infection risk of SARS-CoV-2 in the Wells-Riley model, the number of infectious quanta $${n}_{quantum}$$ must be linked to clinical data. If the number $${n}_{quantum}$$ of infectious quanta inhaled grows linearly with the number ($${n}_{virion})$$ of virions inhaled, these variables could be connected as follows^36^:

13 $$\begin{array}{c}{n}_{quantum}=\xi {n}_{virion} ,\\ \end{array}$$

where the dimensionless coefficient ($\xi )$ characterizes the virus’s transmissibility, i.e., the average number of virions that must be inhaled for infection to occur.  $HI{D}_{50}$ is used to express this median human infectious dose, defined as the dose of virus required to infect $50\%$ of the population, as determined using the Well-Riley model.

14 $$\begin{array}{c}P=0.5=1-{e}^{-\xi HI{D}_{50}},\end{array}$$

and so $\xi$ is related to $HI{D}_{50}$ as

15 $$\begin{array}{c}\xi =\frac{\mathrm{ln}2}{HI{D}_{50}}.\end{array}$$

Despite the lack of $HI{D}_{50}$ data for SARS-CoV-2, data on transmissibility is available in the form of the median tissue culture infectious dose $TCI{D}_{50}$, which is defined as the virus dose required to infect 50% of tissue cultures in a laboratory^37^. A lower $TCI{D}_{50}$ value means fewer virions are needed to cause infection, resulting in a more infectious virus. The human immunodeficiency virus (HIV) has a $TCI{D}_{50}$ unit of roughly 116,000 virions^38^ whereas the influenza A virus has a unit of 1000 to 6000 virions with a mean value of 4000^38–40^. The $TCI{D}_{50}$ data for SARS-CoV-2 was only recently released, and it showed a wide range of values (between 10^3^ and 10^5^ virions) depending on the virus strain^41–43^. These statistics should become more accurate as more trials are completed, but significant disparities in transmissibility across SARS-CoV-2 variants, comparable to viral load, may persist. The $\xi$ can be stated as follows if the in-vitro $TCI{D}_{50}$ index and the in-vivo $HI{D}_{50}$ index have a linear connection^6^.

16 $$\begin{array}{c}\xi =\frac{\mathrm{ln}2}{\delta TCI{D}_{50}}\end{array}$$

The coefficient represents the $HI{D}_{50}$/ $TCI{D}_{50}$ ratio. For influenza, the $HI{D}_{50}$ unit is reported to vary from 1 to 126 $TCI{D}_{50}$ units, with a median value of $5$; or non-immunized patients, it ranges from 0.5 to 3^44^. Wölfel et al.^45^ examined a COVID-19 patient’s sputum during the early stages of the pandemic and found that viral shedding was substantial during the first seven days, followed by a progressively dropping viral load in the following next 21 days. For four weeks, the patient’s sputum average viral load was $7\times {10}^{6}$(RNA) copies/mL, with a maximum of $2.35\times {10}^{9}$ copies/mL. The number of virions is roughly equal to the number of RNA copies^42^. In the present study, the average value of $7\times {10}^{6}$(RNA) copies/mL is used to calculate the viral load.

**Figure 9:**
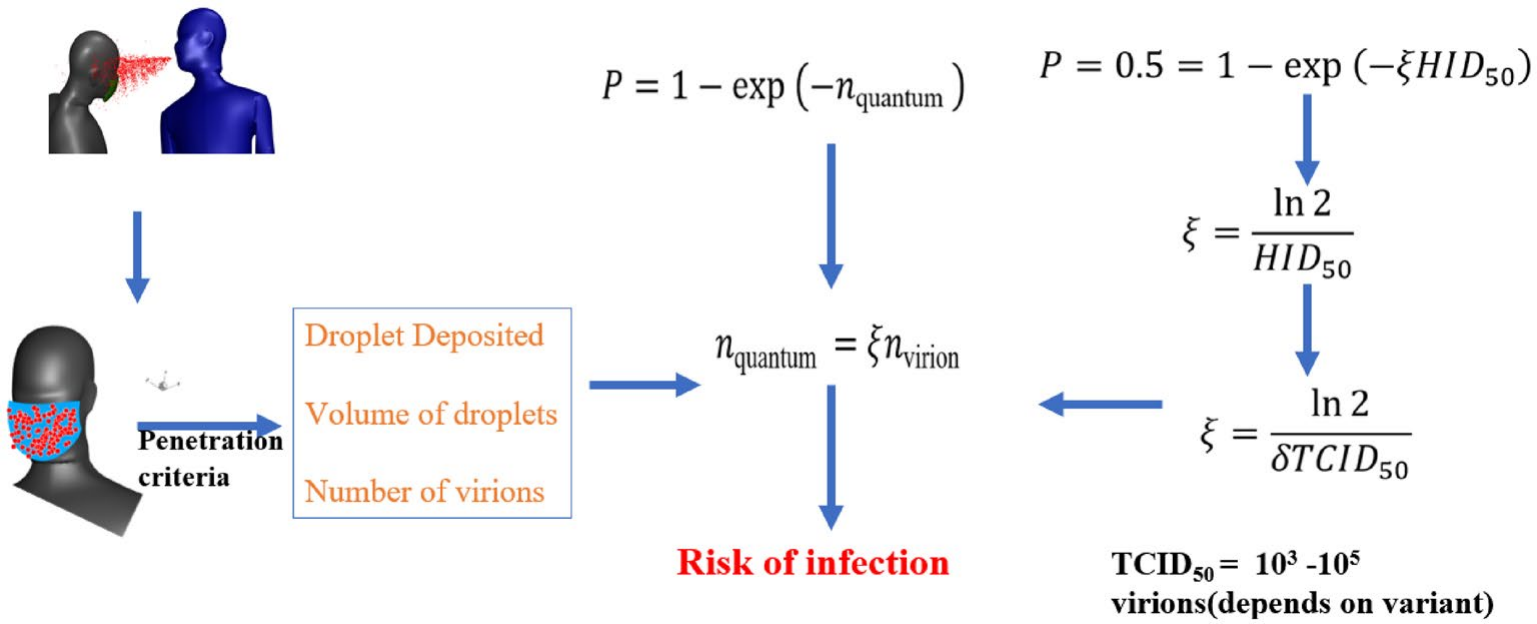
Schematic of risk of infection calculation from droplet deposition.

To calculate the probability of infection and to find the number of virions inhaled by a healthy person, the procedure is shown in Fig. 9. The data from simulations of different ACH and face shield cases have been extracted to calculate the volume of droplets inhaled by the healthy person from the present simulations. The number of virions per ml of sputum has been clinically identified from the existing literature, so if the volume of the inhaled droplets is calculated, then the number of virions can be approximated, and the risk of infection can be estimated. From the numerical simulation results, the diameter of the droplet and its velocity have been extracted for the particles deposited onto the mask.

To calculate the number of virions a healthy person inhales, the saliva volume should be estimated, which is accounted for by the number of droplets inhaled. From the droplets which are impacting the mask, some of the droplets whose kinetic energy is many folds higher than the dissipation energy of the droplets when impacted onto the mask will penetrate the mask and are inhaled by the healthy person^5,6^. To calculate the number of droplets penetrated, the mask data from the experimental study is utilized^6^, as shown in Table 4, which specifies the penetration criteria of the droplets into the mask. They have observed that the droplet penetration through the mask depends on the velocity of impact, mask thickness, and porosity. They found that the diameter of the impacting droplet doesn’t significantly affect the penetration.

Table 4 shows the penetration criteria of the mask for different impact velocities of the droplets^6^. From the experimental criteria, we calculated the volume of the droplets that are inhaled by the healthy person, which in turn can be utilized to calculate the number of virions inhaled. From the value of the number of virions inhaled probability of various variants is calculated as tabulated in Table 5. Out of three masks, the three-layer mask is the most efficient in preventing the droplets from penetrating the mask. The probability of infection varies significantly because of the range of $TCI{D}_{50}$ for different variants have different infection rates, so the probability for every case is the minimum to maximum infection risk based on the various variants that are available. So, further clinical trial data is required to estimate the infection risk accurately. Table 5 indicates the number of virions inhaled by the healthcare person for different ACH and mask types. From this data, the infection probability in these settings is evaluated.

**Table 4:** Penetration criteria by scaling analysis for different layered masks^6^.

Droplet impact velocity (m/s)	Single-layer mask	Double-layer mask	Triple-layer mask
10	***27.22***	***4.37***	1.45
7.5	***20.42***	***3.28***	1.09
5	***13.61***	2.19	0.73
3	***8.17***	1.31	0.44
2	5.45	0.87	0.29

*Bold & Italics*: Penetration happens through the mask. Rest of the cases, penetration does not happen.

**Table 5:** Minimum and maximum probability of infection based upon the number of virions inhaled by the healthy person.

ACH	Mask type	Number of virions inhaled
12	Single layer	2.324 × 10^4^
12	Double layer	4.55 × 10^3^
12	Triple layer	8
9	Single layer	2.8352 × 10^4^
9	Double layer	6.742 × 10^3^
9	Triple layer	10
6	Single layer	2.3932 × 10^4^
6	Double layer	4.305 × 10^3^
6	Triple layer	8

**Figure 10:**
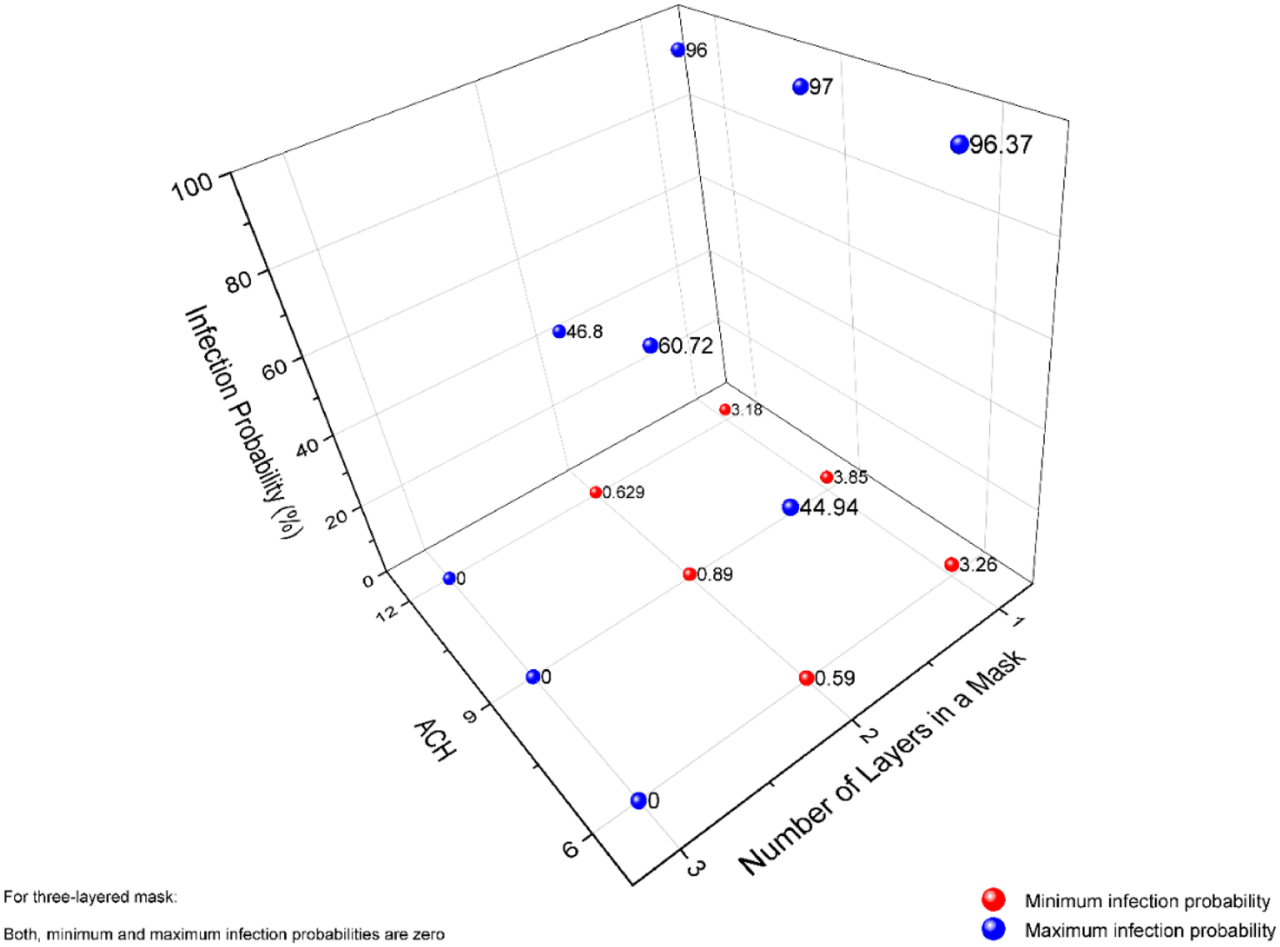
Minimum and maximum infection probability for different layered masks.

The minimum and maximum probability of infection assessed from the simulation results for different layered masks is shown in Fig. 10. The three-layer mask provides the best protection among all the considered masks. In a similar indoor environment, the probability of infection is $0 \%,$ as the number of virions penetrating the face mask is negligible. While for the two-layer face mask, the number of virions inhaled is in the order of ${10}^{3}$ for all ACH rates analyzed in the present study. The probability of the infection varies from a minimum value of $0.59 \%$ to a maximum value of $60.72 \%$. There is a drastic improvement in the infection risk probability for ACH 9 case with a double-layer mask due to flow patterns developed for this particular operating and surrounding conditions. The single-layer mask possesses the maximum risk of infection, as expected. With the single-layer mask, the maximum risk of infection reaches up to $97 \%$ for three different ACH rates considered.

The risk of infection with a different face mask for various ACH rates shows that the increase in the ACH beyond nine in a similar indoor environment may not reduce the infection risk. On the other hand, the increase in ACH rates will require more energy consumption. Therefore, we recommend that the ACH nine is still a good ventilation strategy in similar indoor environments; persons are advised to wear safety equipment like a three-layer face mask and face shield to reduce the risk of infection.

### Resident time of expelled droplets

**Figure 11:**
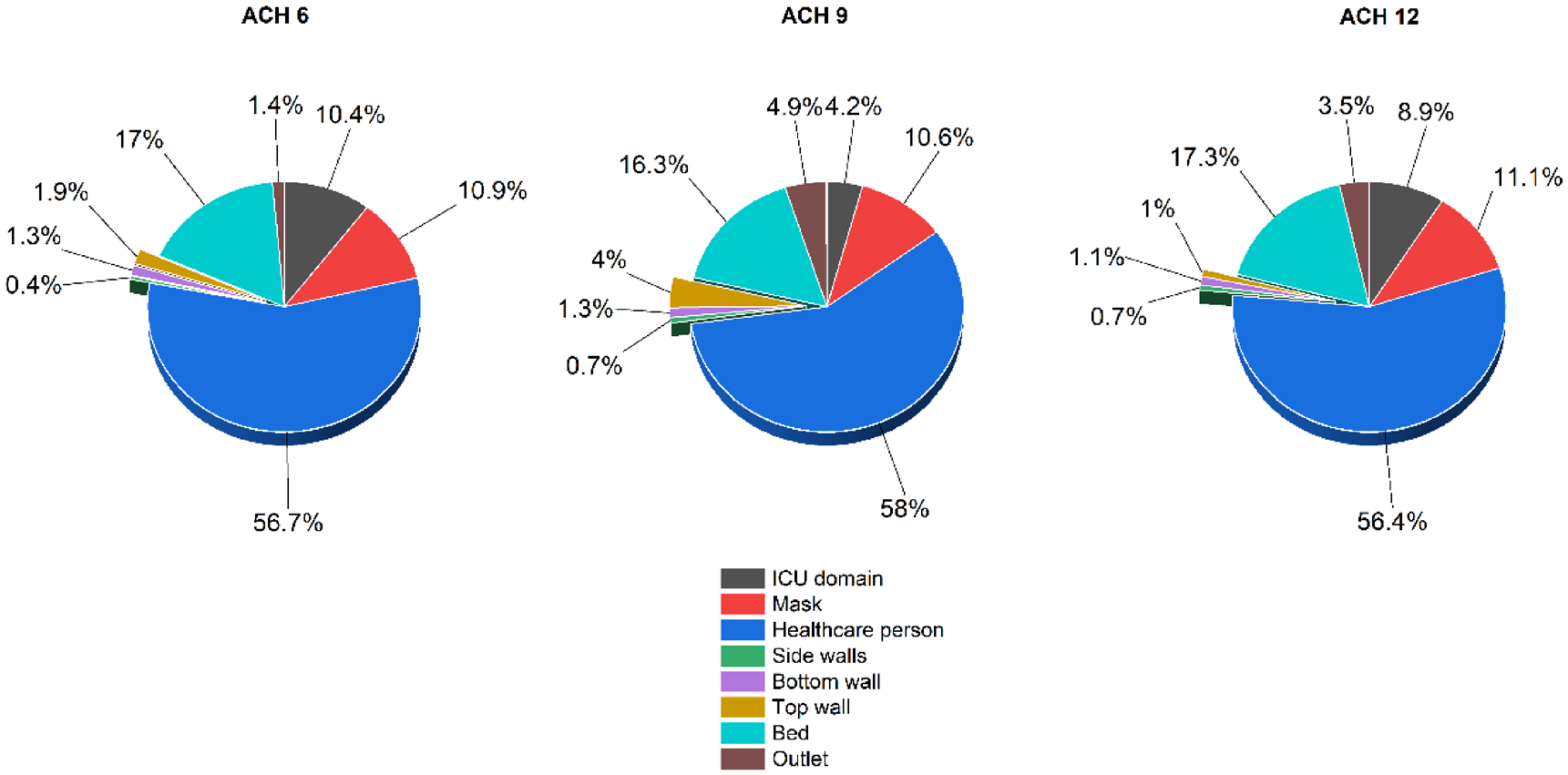
Droplet deposition on the various surface in the ICU room at the end of 30 s.

The resident time of the droplets is defined as the time required for a droplet to achieve its fate since its generation. The quantification regarding the resident time provides insights into the particle removal efficiency of the particular ventilation rate and helps approximate the spread of droplets in the flow domain. In the current study, we have run all the simulations for a flow time of 30 s, and analyzed the fate of the droplets. We have chosen to run it for 30 s since most of the droplets have traveled past the healthcare person within this time. The effect of the change in ACH has been investigated in terms of the droplet extraction in the outlet vent. Figure 11 shows the deposition of the expelled droplets from coughing on various surfaces for different ACH rates. In all three cases, more than 55% of droplets fall on the healthcare person, making the person susceptible to the infection. The results show that increasing ACH from six to nine improves the number of droplets extracted in the outlet vent by ~ 3.5%, while twelve ACH improves it by ~ 2.1%. The amount of particles whose fate is yet to be decided at the end of 30 s, shows how fast and effectively the droplets are being carried out with the ventilation airflow. The percentage of the expelled droplet still moving inside the ICU room at the end of 30 s are ~ 10.4%,4.20%,and 8.9% for ACH six, nine and twelve, respectively. The above data shows that improving the ACH from six to nine results in improved ventilation conditions in the ICU, while ACH twelve performs inferior to ACH nine. Around 10–12% of droplets fall on the mask of the healthcare person, which shows that the change in ACH doesn’t significantly impact the droplet deposition on the face mask in close proximity interaction.

**Figure 12:**
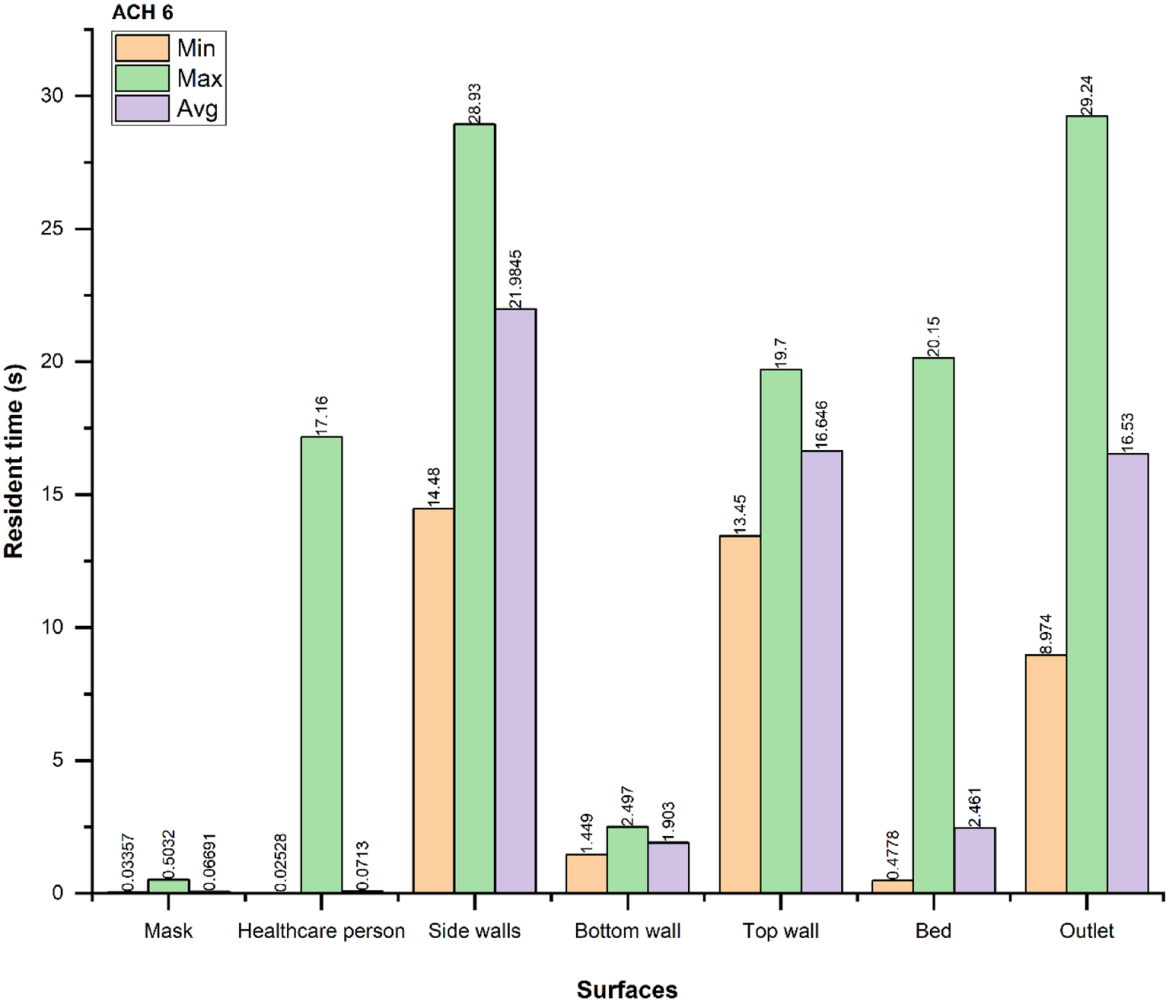
Resident time of the droplets for ACH 6.

**Figure 13:**
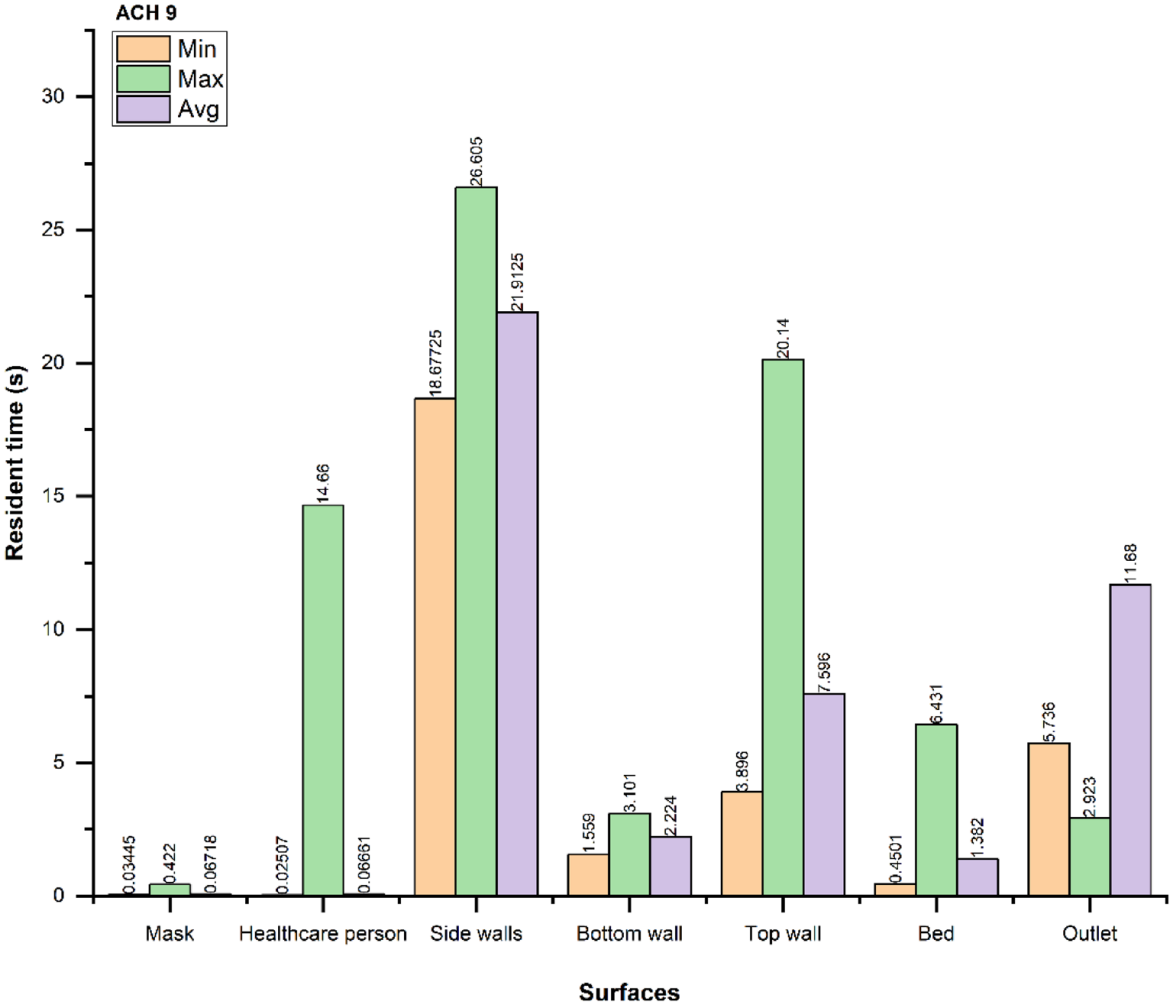
Resident time of the droplets for ACH 9.

**Figure 14:**
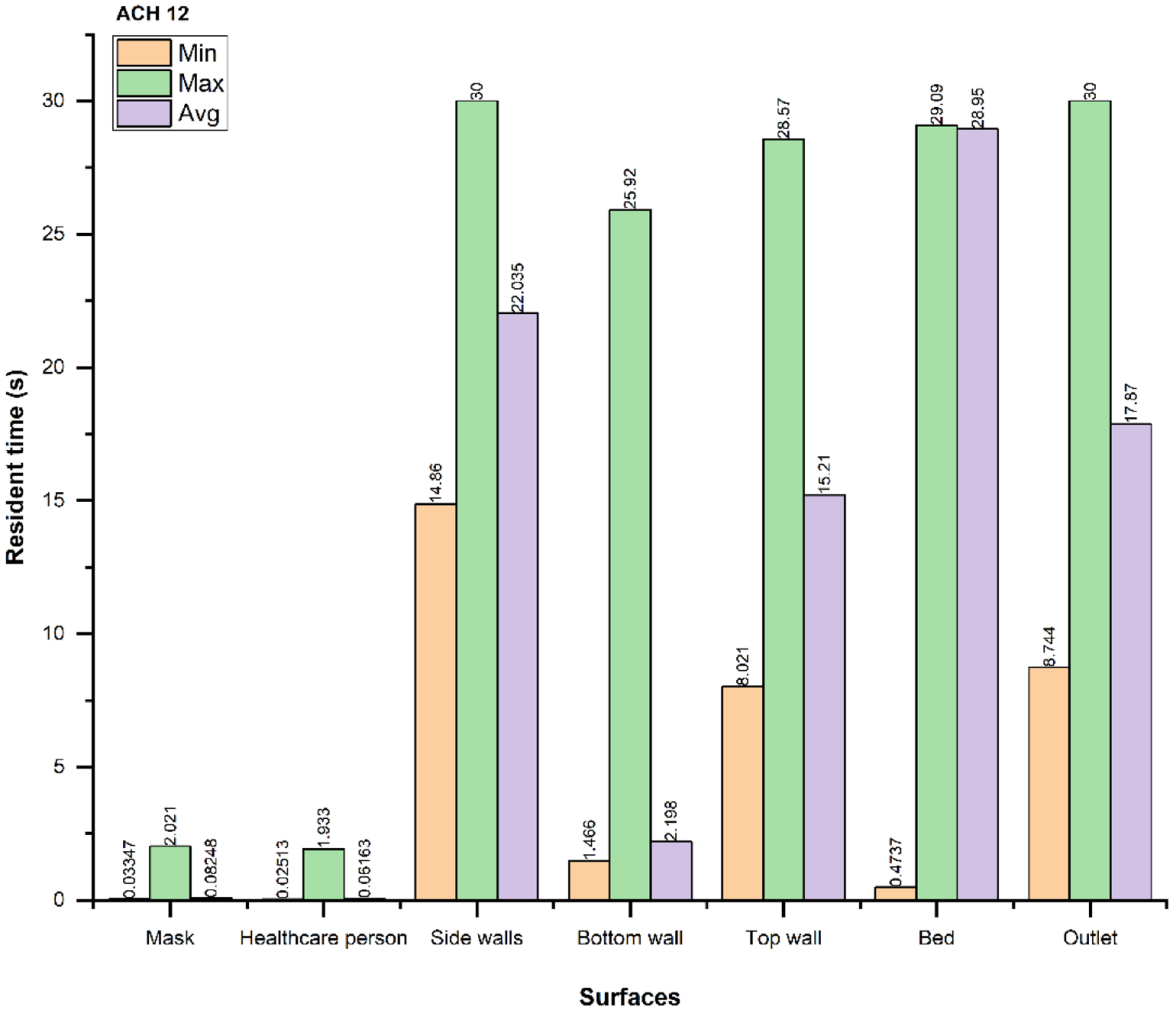
Resident time of the droplets for ACH 12.

Figures 12, 13 and 14 show the minimum (min), maximum (max), and average (avg) time taken for droplets to reach a particular surface. The average time taken for droplets to reach the outlet vent is optimal in the case of ACH 9, while the resident time increases in ACH 12 compared to ACH 6. As shown before, the number of droplets extracted in the ACH 9 case are the maximum out of three cases.

The resident time of the droplets also shows that the average resident time of the droplets is reduced by $\sim 6 s$, when ACH is increased from six to nine. Thus, we recommend using ACH 9 for the larger and faster removal of droplets in similar indoor environments. Increasing the ACH beyond nine to twelve neither improves the droplet extraction nor the resident time of the droplets. Therefore, using ACH twelve is not recommended in similar ICU scenarios from the energy consumption perspective.

## Conclusions

Four different simulations have been carried out with three different ACH rates and one case with a face shield, and the evolution of cough has been analyzed further. The simulation data has been utilized to evaluate the risk of infection to a healthy person. The following conclusions have been drawn.

- The vortex structure generated from the coughing event slowly breaks from 0.1 s due to reaching the peak velocity of the coughing profile and impinges on the face of the health care personnel. The change in ACH has little effect on the vortex evolution and droplet deposition onto the face of a healthy person in proximity.
- Of the three ACH values considered in the study, ACH 9 shows the optimal performance. It offers higher particle removal from the outlet along with a lower resident time of the particles. Further increasing the ACH to 12 doesn’t improve the particle removal efficiency compared to ACH 9 case.
- The requirement of a higher ACH than nine is not recommended in a similar indoor environment considering the extra energy requirement. Instead, it is recommended that the person having a close interaction should wear appropriate personal protective equipment like a face shield along with a face mask.
- The risk of infection for various cases has been calculated and tabulated. The probability mainly depends on the $TCI{D}_{50}$ value of the infectious variant of the respiratory disease; hence most contagious variant has a more considerable value of the risk of infection compared to the least virulent. The data obtained for various virus variants needs to be further determined with greater accuracy to calculate the risk of infection accurately.
- The triple-layer face mask provided maximum protection from the infection, as the probability of infection is zero in similar scenarios. While the probability of the infection risk is up to $97 \%$ in the single-layer mask. Thus, the person in close proximity of the infected person should wear a triple-layer mask and face shield as a precautionary measure.

The study’s limitations are that no coalescence, breakup, and interaction of droplets are considered. The breathing is not included, as well as the mask is modeled as a solid surface. The amount of mass of droplets deposited while atomization of droplets is ignored in the current scenario, which can be quantified and considered in future studies. Although the ACH rates have shown no significant effect on the risk of infection directly to persons present inside, a few previous studies (Abuhegazy et al. 2020; Wu et al. 2021) indicate that the increase in the ventilation rates accelerates the extraction of pathogens from the indoor environment. Therefore, an optimum value needs to be found for specific indoor conditions considering the risk of infection and energy consumption perspective.

## Data Availability

The datasets used and/or analysed during the current study available from the corresponding author on reasonable request.
